# Learning before leaping: integration of an adaptive study design process prior to initiation of BetterBirth, a large-scale randomized controlled trial in Uttar Pradesh, India

**DOI:** 10.1186/s13012-015-0309-y

**Published:** 2015-08-14

**Authors:** Lisa Ruth Hirschhorn, Katherine Semrau, Bhala Kodkany, Robyn Churchill, Atul Kapoor, Jonathan Spector, Steve Ringer, Rebecca Firestone, Vishwajeet Kumar, Atul Gawande

**Affiliations:** Ariadne Labs, Boston, MA USA; Department of Global Health and Social Medicine, Harvard Medical School, Boston, MA USA; Division of Global Health Equity, Brigham and Women’s Hospital, Boston, MA USA; Women’s and Children’s Health Research Unit, Jawaharlal Nehru Medical College, Belgaum, Karnataka India; Population Services International, Delhi, India; Lao Friends Hospital for Children, Luang Prabang, Lao People’s Democratic Republic; Department of Neonatology, Brigham and Women’s Hospital, Boston, MA USA; Population Services International, Washington, DC USA; Community Empowerment Labs, Lucknow, Uttar Pradesh India; Department of Surgery, Brigham and Women’s Hospital, Boston, MA USA; Department of Health Policy Management, Harvard T.H. Chan School of Public Health, Boston, MA USA

## Abstract

**Background:**

Pragmatic and adaptive trial designs are increasingly used in quality improvement (QI) interventions to provide the strongest evidence for effective implementation and impact prior to broader scale-up. We previously showed that an on-site coaching intervention focused on the World Health Organization Safe Childbirth Checklist (SCC) improved performance of essential birth practices (EBPs) in one facility in Karnataka, India. We report on the process and outcomes of adapting the intervention prior to larger-scale implementation in a randomized controlled trial in Uttar Pradesh (UP), India.

**Methods:**

Initially, we trained a local team of physicians and nurses to coach birth attendants in SCC use at two public facilities for 4–6 weeks. Trained observers evaluated adherence to EBPs before and after coaching. Using mixed methods and a systematic adaptation process, we modified and strengthened the intervention. The modified intervention was implemented in three additional facilities. Pre/post-change in EBP prevalence aggregated across facilities was analyzed.

**Results:**

In the first two facilities, limited improvement was seen in EBPs with the exception of post-partum oxytocin. Checklists were used <25 % of observations. We identified challenges in physicians coaching nurses, need to engage district and facility leadership to address system gaps, and inadequate strategy for motivating SCC uptake. Revisions included change to peer-to-peer coaching (nurse to nurse, physician to physician); strengthened coach training on behavior and system change; adapted strategy for effective leadership engagement; and an explicit motivation strategy to enhance professional pride and effectiveness. These modifications resulted in improvement in multiple EBPs from baseline including taking maternal blood pressure (0 to 16 %), post-partum oxytocin (36 to 97 %), early breastfeeding initiation (3 to 64 %), as well as checklist use (range 32 to 88 %), all *p* < 0.01. Further adaptations were implemented to increase the effectiveness prior to full trial launch.

**Conclusions:**

The adaptive study design of implementation, evaluation, and feedback drove iterative redesign and successfully developed a SCC-focused coaching intervention that improved EBPs in UP facilities. This work was critical to develop a replicable BetterBirth package tailored to the local context. The multi-center pragmatic trial is underway measuring impact of the BetterBirth program on EBP and maternal-neonatal morbidity and mortality.

**Trial registration:**

Clinical trials identifier: NCT02148952.

## Background

Reducing the quality gap between the knowledge of effective interventions and care delivered often takes complex interventions requiring change in individual behavior, leadership, and systems [1]. However, rigorously testing the effectiveness of these types of quality improvement (QI) interventions through randomized controlled trials in real-world settings is challenging, particularly where control of key contextual factors is limited [2]. Pragmatic and adaptive trial designs are increasingly being used to study QI interventions to provide the strongest evidence for impact prior to broader scale-up [3–7]. Integrating these approaches into the overall study design ensures ongoing learning on where local adaptation of the intervention or strategy processes is needed before or during the implementation of the trial [8].

Unacceptably high rates of maternal and neonatal morbidity and mortality persist in many resource-limited settings (RLS), despite efforts to achieve the Millennium Development Goals through increasing uptake of facility-based deliveries [9–11]. Much of this suffering is preventable, yet there remains an implementation gap between what we know works and the care received by women in labor and their infants in these facilities [12, 13]. Ensuring that these women and infants receive essential birth practices (EBPs) known to prevent or manage complications during facility-based deliveries is a critical step towards achieving the needed reduction in harm [14]. However, to achieve large-scale impact, effective scalable solutions are needed. The World Health Organization (WHO) Safe Childbirth Checklist (SCC) is designed to help birth attendants remember EBPs at four critical pause points (PP) in the delivery process: (1) at admission, (2) just prior to delivery, (3) in the immediate post-partum period, and (4) prior to discharge [15]. This tool, when implemented effectively, has the potential to contribute to ongoing work to improve facility-based quality through scalable solutions and reach the goals of improved maternal and neonatal health. The theoretical framework underlying the implementation approach was based on the successful design of the Safe Surgical Checklist interventions [16]. This work found that success required the following: (1) leadership engagement and commitment, (2) focused introduction of the checklist to end-users including understanding of existing quality gaps and benefits in addressing preventable causes of harm, (3) support through coaching to ensure ongoing use and sustainability, and (4) ongoing monitoring and feedback on intervention uptake and behavior change.

Previously, we showed that an on-site coaching intervention focused on the use of the SCC-improved performance of EBPs in a single facility in Karnataka, India [17]. Some of the contextual factors critical to success included the strong systems in the hospital (adequate supplies and staffing), strong on-site leadership committed to improvement, which provided coaching to the front-line birth attendants, and a local SCC champion.

We planned to test the ability of the SCC program to not only change behavior but also save lives by studying the impact using a pragmatic and adaptive randomized controlled effectiveness trial design in a larger number of facilities in Uttar Pradesh. We chose a pragmatic study design to determine if change in behavior can lead to reduction in mortality and morbidity in a “real world” setting. We also recognized that, similar to other complex interventions tested in resource-limited settings, there would be important contextual factors in Uttar Pradesh (UP) such as variability in on-site leadership to champion the SCC and gaps in supplies and staffing that could limit the ability to change birth practices. Therefore, an adaptive design was incorporated to allow for the context-driven changes in implementation that would likely be needed.

We included an adaptive design approach during the learning phase to test and modify aspects of the intervention package prior to scale-up through the large-scale trial. The BetterBirth toolkit was designed to contain all of the components identified as critical for replicating effective programs including an implementation package, training, facilitation (coaching), evaluation, and plans for sustainability [4]. The adaptation focused on ensuring that the core functions remained, but how they were implemented (the processes) reflected the contextual factors of the sites in Uttar Pradesh. The intervention was designed to be scalable within existing systems and so provides no clinical skill training or supplies. Following adaptive study design, the process for refining the toolkit was guided by performance measurement of behavior change and by qualitative feedback on feasibility, contextual barriers, and acceptability during revision-and-testing cycles prior to full study rollout. Here, we describe the process and results of intervention adaptation to create a replicable package designed to drive behavior and system change prior to launching the larger pragmatic randomized control trial.

## Methods

### Study aims

The primary outcomes of this adaptive phase of the study was the successful adaptation of the intervention design resulting in change in essential birth practices in the pilot facilities prior to scale-up in the randomized control study. The secondary outcome was to describe the contextual factors and resulting changes to facilitate any further context-related adaptations identified as needed during the full-scale trial.

### Study sites

Uttar Pradesh is a state in Northern India. Rates of maternal and neonatal morbidity and mortality are high, with estimated maternal mortality rates of 258/100,000 live birth and neonatal mortality of 49/1000 [18]. Auxiliary nurse midwives and staff nurses provide the majority of delivery services, supported as needed by the head of the facility, typically a physician for complicated cases. Most health centers do not provide full management of these cases but provide Basic Emergency Obstetric and Neonatal Care (BEmONC) with referral to a district women’s hospital equipped to provide Comprehensive Emergency Obstetric and Neonatal Care (CEmONC) including Cesarean sections.

Following initial adaptation of the intervention as tested in Karnataka, the BetterBirth program (phase I) was implemented in two health center-level facilities. After the second adaptation, we re-tested the intervention in two health centers and one district women’s hospital (phase II). Based on lessons learned from phase I, only facilities with a minimum of three trained nurse birth attendants and observation data were eligible for inclusion in results for phase II.

### The checklist

The WHO Safe Childbirth Checklist format (four pause points: admission, immediately before delivery, within 1 h post-delivery, and before discharge) was unchanged, but content was modified to reflect national guidelines and local context [17, 19].

### Intervention package phase I

The initial BetterBirth package was built on the Karnataka study intervention approach with adaptation to reflect identified contextual differences in Uttar Pradesh [17]. For example, in Uttar Pradesh, there was no pre-existing leadership at facilities for the intervention, weaker supply chain for essential birth supplies (EBS), and lower staffing levels (Table 1). Modifications included explicit engagement of facility leadership through site visits and educating staff involved in childbirth in a 3-day training focused on the burden and causes of maternal and neonatal morbidity and mortality and the use of the checklist to ensure delivery of essential birth practices. The training concluded with an official launch of the checklist including hanging of large posters of each pause point at the location where the specific birth practices were provided. Checklist use was then supported by a coaching team led by a physician and including nurses trained in delivery practices. The team visited the facilities every 1 to 2 weeks for 4–6 weeks to encourage uptake by birth attendants and help them identify and address barriers preventing their performance of the EBPs. Focusing on sustainability, supplies were not provided as a part of the intervention.

**Table 1:** Adaptation to create the BetterBirth (BB) intervention package used in the randomized control trial

	Karnataka Pilot	First adaptation	Second adaptation	RCT
Leadership engagement	Study lead introduced to district and facility leadership	Non-standardized introduction to district and facility leaders	Formalized introduction at district and facility including strong focus on motivation to drive adoption	Same as in phase II
Education of facility staff	1-day training on the SCC supported by instructional video, and hands-on simulation	3-day training for staff (2 days didactic, 1-day coached practice using the SCC)	Semi structured launch including 1–2-day workshop introducing SCC, problem solving, and strong focus on motivation including video and anthem	Structured 2-day launch with increased focus on implementation of the SCC with day 2 on-site for official start
Coaching support	Core team of head of the hospital and senior physician and labor nurse supplemented by physician from the study team	Physician-led team of physician and nurses coaching birth attendants	Peer-to-peer model: Nurse coaches for birth attendants (behavior change), physician coach facility leader (systems change and SCC leadership), and childbirth quality coordinator	Same with additional focus on district lead to build support for SCC
	Coaching provided during normal clinical routines supplemented every 2 weeks by study physician	Coaching provided Every 1–2 weeks for 4–6 weeks		Coach training using standardized curriculum focused on coaching skills to drive behavior change and barriers framework (opportunity, ability, motivation) with strong focus on motivation
	Coach training through review of SCC	Coach training through 2-day, on-site workshops focusing on clinical skills	Coach training focused more on QI approaches and behavior change	
Data feedback loop	Subset of baseline observation data feedback to staff to identify quality gaps	None	Paper-based system used to capture and review observation data by coaching team to identify persisting gaps and behavior change. Apps used to capture study-related data	Robust app-based system to provide real-time data feedback on coach observations and essential birth supplies to BB team, facility, and district. All study data continued to be captured by existing apps
Safe birth supplies (SBS) availability	Largely available	Supply chain gaps	Increased focus of coach TL to help the facility head and district leaders leverage existing resources to address gaps	Strengthened focus for coaching and advocacy at facility and district levels for strengthening EBS availability

*TL* physician coach team leader, *EBP* essential birth practices, *RCT* randomized control trial

### Data sources

#### Essential birth practices observation

Trained nurse observers captured birth attendant behavior of EBPs and use of the SCC during the first three of the SCC pause points using a standardized data collection tool. Data from the EBP observation was collected on paper and entered into a database. Pre-intervention data were collected prior to the coaching intervention for approximately 4 weeks. Post-intervention data were collected from 4 to 12 weeks after the coaching intervention was initiated. The trained nurse observers did not interact with the birth attendants or patients before, during, or after the observations.

The initial phase was modeled on a quality improvement small test of change, so no initial sample size was used. For phase II, observations were done for 4–8 weeks to limit the duration of coaching being evaluated. Using a post hoc power calculation, in the initial adaptation, we had 80 % power to detect a 25 % change in behavior. In the second adaptation, we had 80 % power to detect a 10 % change in behavior.

### Qualitative data

#### Better birth program documents

Documentation of modifications made to the intervention was gathered through review of BetterBirth program documents. These included the study protocol and modifications, reports to Scientific Advisory Board, and the coaching visit tracker. Interviews with coaches, facility staff, and the implementing team were conducted to identify priorities and barriers as part of program improvement.

### Data analysis

Interview results were used to prioritize areas where intervention change was needed. Program documents were reviewed, and the implementation team interviewed to confirm changes made during the adaption phases. Prevalence of performance of each EBP was calculated. Chi-square test was used to measure change in performance in phase II pre/post-coaching using chi-square test adjusting for clustering by facility. Analysis was conducted using SAS v9.3.

### Human subjects

The study was approved by the Harvard T.H. Chan School of Public Health IRB, World Health Organization Research Ethics Review Committee, Indian Council for Medical Research, Jawaharlal Nehru Medical College Institutional Ethics Committee on Human Subjects Research, Lucknow Ethics Committee, and PSI Research Ethics Program. Written informed consent was obtained before observation.

## Results

### Results from phase I

**Fig. 1:**
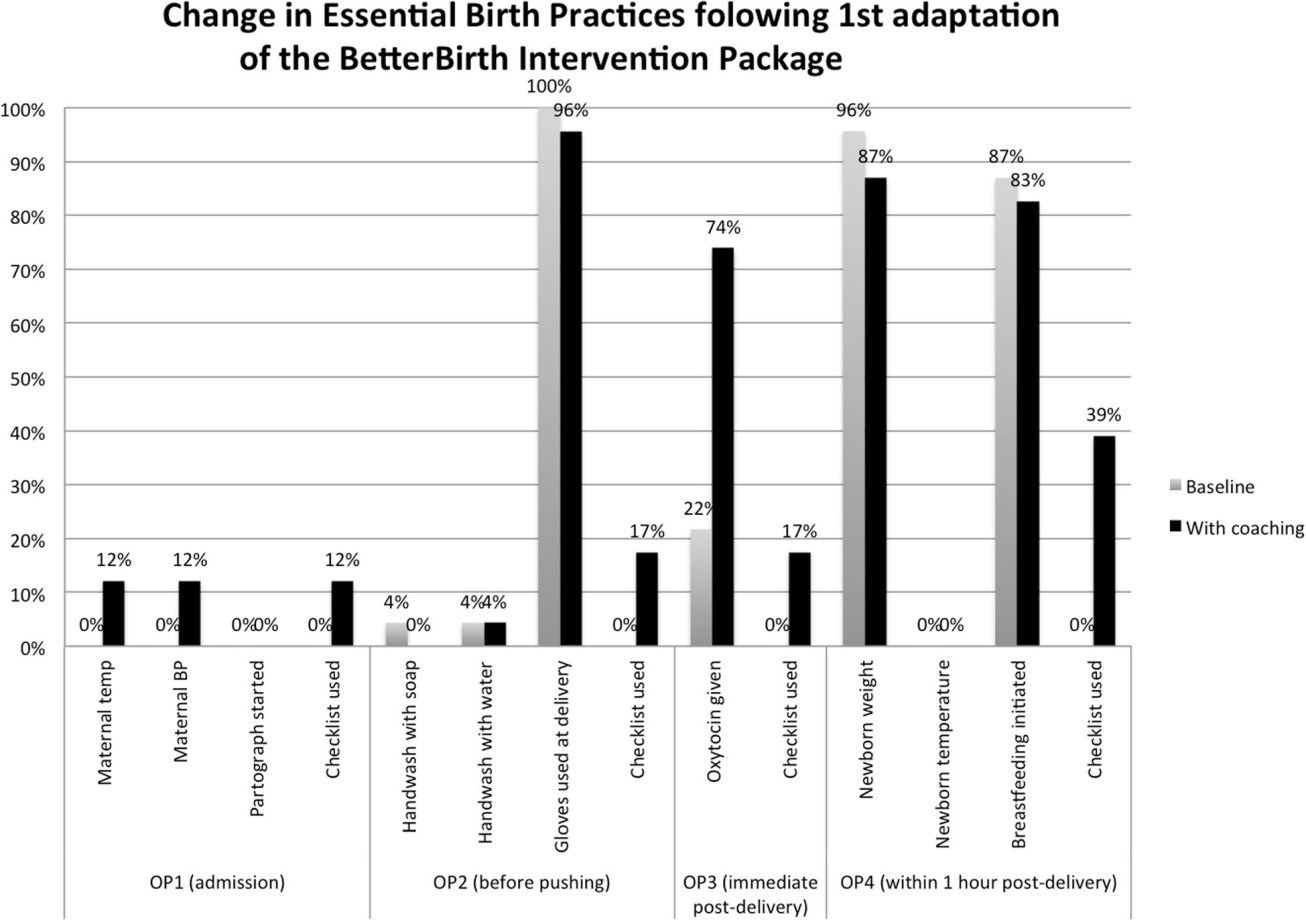
Change in observed essential birth practices performed by birth attendants following implementation of the BetterBirth Program in the first two facilities in Uttar Pradesh following initial adaptation. Trained observers collected data at four predetermined observation time points (*OP*s) during the perinatal process (OP1: at admission; OP2: before pushing; OP3: immediate post-delivery; OP4: within 1 h post-delivery). Numbers observed OP1: pre 20, post 33; OP2: pre 23, post 23; OP3: pre 23, post 23; OP4: pre 23, post 23

After introduction of the physician-led coaching and use of checklist in two phase I facilities, the site staff reacted positively to the concept of the checklist and its role in preventing harm and improving quality. However, system (staffing and supplies) and persistent motivational barriers were associated with limited observed behavior change (Fig. 1). The only substantial improvement across the labor and delivery period was seen in appropriate delivery of oxytocin immediately post-partum (22 to 74 %), with the SCC used between 10 % (at admission) and 39 % (within an hour of delivery) of observed care interactions. Based on discussions with coaches and study staff as well as observations of activities, a number of needed adaptations were identified. While engagement of the heads of the facilities was important, higher level engagement up to the district level and ongoing coaching at the facility management level were needed to address identified system-level issues such as supplies and equipment. Physicians also faced challenges in being effective and accepted coaches for nurses and auxiliary nurse midwives who comprised the overwhelming majority of the trained birth attendants. This was felt by the team as due in part to hierarchal rather than partnership interaction between physicians and nurses in the coaching relationship. Finally, the dose of coaching visits and strategy was inadequate to move beyond knowledge change to drive the needed behavior change to use the checklist and deliver essential birth practices.

### Adaptation for phase II

Based on this lack of improvement in phase I, a series of revisions were made to the BetterBirth intervention package (Table 1). Revisions focused on a more standardized three-phase approach from initial engagement, through launch of the program, and ongoing support (engage, launch, support). A more formal engagement approach was implemented with a meeting with the district chief medical officer to provide information on the goals and procedures of BetterBirth program and gain commitment to support any system-level changes. This engagement visit was followed by a similar visit to intervention facility heads. The program also incorporated a motivational strategy anchored in enhancing professional pride and effectiveness. This motivational strategy started during the 2-day launch training and included a video with a motivational theme and anthem, as well as a video that demonstrated checklist use, featuring an experienced birth attendant and a new nurse who use the checklist to ensure quality care. Facilities also had to have a minimum of three trained nurse birth attendants to ensure that targeted providers were available during coaching visits.

As part of the support phase, we formalized provider, facility, and district leadership engagement and shifted to exclusively nurses coaching birth attendants and support staff in appropriate tasks. Recognizing that coaching was needed at the leadership level as well, physician coaches focused on facility leaders to engage and ensure local ownership and address the facility-level contextual factors, such as inadequate safe birth supplies that could prevent behavior change. Coaches also worked with the leader to designate a facility Childbirth Quality Champion; the quality champion was mentored to develop into a facility-based coach to ensure sustained SCC use and associated quality improvements. The coaching intervention was also intensified including the following: visits planned for 2–3 times per week, incorporation of an observation tool to focus coaching on checklist use and EBP and document challenges, and feedback of data from the observation tool from coaches to their team leaders to address the challenges.

### Results from phase II

**Fig. 2:**
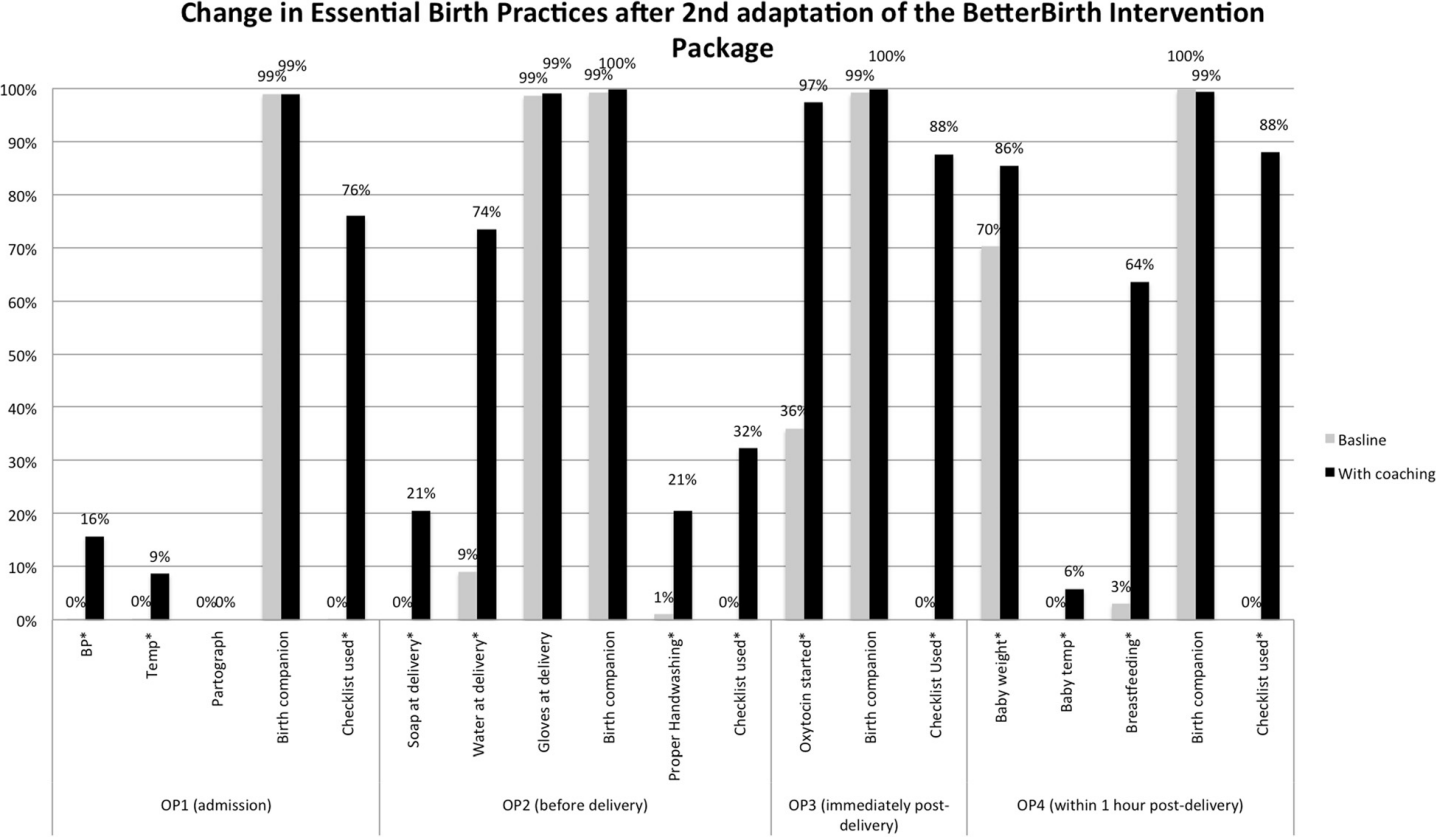
Change in observed essential birth practices performed by birth attendants following implementation of the BetterBirth Program in three facilities in Uttar Pradesh after the second adaptation. Trained observers collected data at four predetermined observation time points (*OP*s) during the perinatal process (OP1: at admission; OP2: before pushing; OP3: immediate post-delivery; OP4: within 1 h post-delivery). Numbers observed per observation point (*OP*). OP1: pre 624, post 335; OP2: pre 521, post 402; OP3: pre 523, post 403; OP4: pre 522, post 409. *p < 0.001. Rates are adjusted for clustering by site

After intervention modification from phase I, we initiated phase II in three facilities with >3 trained nurse birth attendants and conducted two to three visits per week from the nurse coaches, totaling 15–18 coaching visits per facility (Fig. 2). Physician coaches accompanied the nurse coaches in approximately one quarter of visits at the health centers and one half of visits at the district women’s hospital. Trained observers collected data similarly to data collection in phase I.

In this adapted model and adjusting for clustering by site, we saw significant improvements in a number of critical areas including the following: screening for pregnancy-associated complications (maternal blood pressure, 0 to 16 %, and temperature measured, 0 to 9 %); infection control (proper hand hygiene, 1 to 21 %); oxytocin immediately after delivery (36 to 97 %); and post-partum care of the newborn (weight taken, 70 to 86 %, and breastfeeding within 1 h of delivery, 3 to 64 %), all *p* < 0.001. Checklists were used in 76 to 88 % of the pause points observed.

### Adaptation prior to the randomized control trial

Despite an increase in behavior change in phase II, a number of adaptations to further strengthen the intervention were instituted largely in the support activities prior to launch of the full trial, reflecting further lessons learned (Table 1). Feedback from the facilities and coaches identified that less frequent weekly visits, but engagement over a longer period of time, were likely more effective to continue to build on the coach-coached relationship and provide time for change of more resistant behaviors. As a result, the coaching visits in the randomized control trial will start at twice a week and wean down to end over 8 months. To increase the potential for sustainable and ongoing change, we also increased the capacity building of the facility-based Childbirth Quality Champion who will provide ongoing coaching and champion further quality improvement in maternal and neonatal care at the facilities. Extending the duration of the coaching intervention with a decrease in intensity over time allowed for a transition period to coaching by the Childbirth Quality Champion without a large increase in coach resources needed.

Building on experience from team members and publically available curricula in coaching for behavior change, we developed a short practical workshop for coaches in developing the needed skills to engage the nurse birth attendants and teach the attendants how to identify and address barriers and care about change. The workshop also provided physician coaches skills to coach the head of the facility focusing on the needed leadership and motivation for system changes and to mentor the facility Childbirth Quality Champion.

We also strengthened the data feedback loop expanding the app-based system designed for research data collection to include electronic capture for rapid feedback of coach-reported EBP practices to the facility and district. This system was also used by the coaching team to identify individual coaching challenges to address and successes for sharing to strengthen the coaching intervention and identify system-issues for escalation to the physician coaches. The physician coach will use the data to motivate leaders at the facility, district, and state levels to understand and drive system improvements and celebrate successes. Finally, an explicit advocacy component was added to coach and coach team leader work designed to activate leadership at the district and state levels to ensure a strong supply chain and harmonization of the BetterBirth intervention with the national quality assurance program and guidelines.

## Discussion

### Effectiveness of the adaptive design process

Using an iterative process to test and adapt a proven effective intervention to reflect local contextual factors and results of measurements, we were able to develop a SCC-based coaching package able to drive behavior change in essential birth practices in primary and community health centers in Uttar Pradesh. The final intervention design, which will be tested in the fully randomized control trial, works through two processes: (1) producing direct provider practice change and (2) igniting and enabling effective health system response through strengthened supervision, supplies, and norms working at the facility levels. We were able to develop an effective intervention package through an approach that incorporated adaptive study and quality improvement methodologies [20-22]. We used quantitative measurement of behavior change and contextual factors combined with qualitative capture of identified barriers to strategically drive modifications of the intervention. Changes were then re-tested to ensure that the approach was appropriate to the realities of the facility and area targeted for the full trial and further refinements needed as indicated. This approach has been important in ensuring that interventions which focus on changing behavior when replicated in new settings with important contextual differences are appropriately adapted and formalized prior to large-scale testing [5].

### Strengthening the intervention

A number of the adaptations we made to strengthen our intervention strategy are supported by evidence in other work focused on driving behavior change and improvement in health care. During the first adaptation, the coaching focused on identified gaps of skills and supplies as the major barriers to providing EBPs, reflecting similar challenges in other parts of India [22, 23]. However, we recognized that motivation to adopt change was an equally important barrier based on feedback from coaches. The importance of motivating healthcare workers to care about quality and be willing to improve has also been described in other work focused on changing behavior in care delivery [24–26]. In response, the coaching approach was adapted to focus on diagnosing the barrier to change using a social marketing framework opportunity (such as supplies, ability, or motivation) and provide coaching to address any of the underlying causes. [27, 28] The motivational strategy also included developing videos shown during the facility launch of the BetterBirth intervention to increase the birth attendants’ belief in the potential for improvement, the role of the SCC, and their own importance in reducing maternal and neonatal harm.

The effectiveness of peer-to-peer coaching to change behavior and drive improvement has also been described in other settings. In Rwanda, the MESH program has been effective using nurses to coach front-line providers resulting in improvements in quality in maternal care and other clinical spheres [29, 30]. Peer-to-peer coaching at the physician level was also strengthened, adapting the role of the physician coaches to focus on stronger leadership engagement at multiple levels to build local ownership and leadership to make the system changes needed to address barriers not within the control of the nurse birth. This approach has been successful in interventions that focus on leadership and management as a critical component to driving and sustaining system-based quality improvement [31]. For the coaching to be successful, skills needed include communication, diagnosing and overcoming resistance, problem solving, building relationships and debriefing through feedback of observed behavior, and other performance data [32]. Recognizing these needs, we ensured that the coaches have the necessary coaching skills as well as the content knowledge. These materials will be used to train new coaches in the study and serve as a resource for other programs interested in implementing a SCC-based program [19].

While we saw a large improvement in uptake of EBPs after the second round of adaptations, there remained areas more resistant to change. These included components of maternal and neonatal vital sign measurement, partograph use, and placing the newborn on the mother’s abdomen (skin-to-skin) post-delivery (only observed at one facility with increase from 0 to 28 %). Some of these behaviors have been described in other settings as challenging to change and may reflect the ability to observe immediate benefit or an earlier stage of behavioral change along the pathway needed for consistent performance [26, 33]. In response, we further strengthened the coaching methodologies and the focus on engaging leadership as noted above.

Further adaptations to the intervention were focused on identifying and addressing the needed system change and planning for long-term sustainability. Feedback of monitoring data supported by coaching to develop and implement action plans has been shown to be an important strategy to drive system improvement [34]. For the randomized control trial, we expanded the app-based component to provide real-time data on observed behavior change and system challenges based on the coaching observations. These apps were tested in the pilot sites after study observation had concluded to ensure feasibility and acceptability. These data will be used by the physician coaches to strengthen nurse coaching and coach on utilization of these data by the facility and district leaders to identify and address challenges including gaps in the needed safe birth supplies and celebrate successes. We explicitly opted to not supplement birth supplies reflecting our goal of planning for sustainability from the start of the intervention. We strengthened the advocacy strategy to further spur system changes at the district and state levels and to ensure that the intervention remains aligned with the NRHM’s quality assurance management system.

The need for longer-term support when implementing a behavior change intervention is similar to experiences with other efforts and is consistent with the recognition of the need to expand the CDC’s Replicating Effective Programs Framework to include facilitation or coaching beyond short-term technical assistance [4, 35]. Consistency of our approach with the CDC framework which designed to accelerate the effective scale-up of programs in new settings will increase our potential to overcome the common challenge of effective scale-up as the study rolls out [4]. To address this long-term need, we integrated a greater focus on developing the facility-based Childbirth Quality Champion to continue to drive the improvements through coaching and motivation to use the SCC beyond the end of the study for sustained change.

### Limitations

Our study has a number of limitations. The performances measured could have been influenced by having an observer present [36]. However, because the same approach was used both at baseline and after coaching, the effect would likely have reduced observed change. Our changes were based on the experiences of a small number of facilities, and so our conclusions and adaptations may not be applicable to the broader range of facilities in UP. However, we used a mixed methods approach to include both observed changes in performance as well as feedback from the coaches and program implementers to bring in contextual factors into our adaptations to reduce this risk. The repeated measurements, integral in an adaptive trial, could have increased the risk of a type 1 error. Finally, secular trends may have altered performance EBPs as the Government of India has adopted a focus on quality improvement. The planned randomized control trial will be able to test if the new package is effective in improving EBPs as well as clinical outcomes independent of this potential limitation.

## Conclusions

This process of improvement through measurement and iteration was critical to adapt the intervention from a single larger facility in Karnataka with strong internal commitment and motivation to smaller, less well-resourced facilities in UP lacking pre-existing leadership. Many of the changes reflect evidence in the literature, but the testing and adaptation were critical to understand which practices should be added to the strategy and how to incorporate them into a replicable package. While this approach to adapting the intervention package to address contextual differences in a new setting and the challenges of larger-scale implementation took time, it resulted in a package able to result in behavior change even in smaller facilities with multiple challenges. Implementation of this adapted package will ensure that the randomized control trial will be able to measure if change in behavior and systems results in the targeted impact on maternal and neonatal harm.
